# Right ventricular function among South East Nigeria children with sickle cell anaemia

**DOI:** 10.1186/s12887-020-02143-4

**Published:** 2020-05-21

**Authors:** Josephat M. Chinawa, Bartholomew F. Chukwu, Awoere T. Chinawa, Edmund N. Ossai, Anthony N. Ikefuna, Ann E. Aronu, Egbuna O. Obidike

**Affiliations:** 1 Department of Paediatrics, College of Medicine, University of Nigeria Enugu Campus, Enugu, Nigeria; 2 Consultant Community Physician and Lecturer Enugu State University Teaching Hospital, Enugu State, Enugu, Nigeria; 3 Department of community Medicine College of Health Sciences Ebonyi State University, Abakaliki, Nigeria

**Keywords:** Anaemia, Children, Enugu, Sickle cell anaemia, Right ventricular function

## Abstract

**Background:**

Sickle cell anaemia (SCA) is characterized by attendant ischemia-reperfusion injury especially to the heart.

**Methods:**

The aim of this work is to compare the right ventricular function of children with SCA in steady state (subjects) with those with haemoglobin AA genotype (controls), using echocardiography. It is a cross-sectional study, which echocardiographic measurements to assess right ventricular function among children with SCA and their controls.

**Results:**

The mean trans annular plane systolic excursion (TAPSE) in subjects, 28.24 ± 5.23 (Z score: 0.258 ± 1.10) was higher than that in control, 25.82 ± 3.59 (Z score: - 0.263 ± 0.80), and the difference in mean was statistically significant, (t = 2.703, *p* = 0.008).

Significantly higher proportion of subjects with sickle cell anaemia had right ventricular dysfunction (Abnormal TAPSE), 25 (50.0%) when compared with those in control, 11 (22.0%), {χ^2^ = 8.5, *p* = 0.0035}.

A higher proportion of subjects with sickle cell anaemia (25.5%) had Pulmonary hypertension (RVP) when compared with control (2.0%) and the difference in proportions was found to be statistically significant, (χ^2^ = 11.668, *p* = 0.001). The prevalence of right ventricular diastolic dysfunction in subjects was 9.8% while control was 0%.

**Conclusion:**

Children with sickle cell anaemia present with right ventricular dysfunction. Prevalence of right ventricular systolic and diastolic dysfunction were higher in subjects. More of the subjects in this study (25.5%) had pulmonary hypertension.

## Background

Sickle cell anemia is a hematologic and genetic disease characterized by recurrent episodes of ischemia-reperfusion injury to multiple vital organ systems [1]. Though volume overload is noted as a cause of cardiac abnormalities in SCA, other potential causes are ischemia-reperfusion injury and iron overload toxicity in chronically transfused patients [2]. It is important to note that the underlying haemolytic anaemia in children with sickle cell anaemia increases cell-free plasma haemoglobin which depletes nitric oxide and causes vasoconstriction, leading to pulmonary hypertension [2]. Both systolic and diastolic dysfunction of the right ventricle coupled with right ventricular hypertension had been documented in sickle cell anaemia at age 3 years [2]. They are due to progressive increase in pulmonary vascular resistance, this fact may be one of many contributory factors among several unknown or yet to be discovered reasons.

For instance, the cause of right ventricular dysfunction in children stems mainly from progressive increase in pulmonary resistance, whereas in adults; it is usually from high steady-state serum lactate dehydrogenase (LDH) levels, largely reflecting intravascular haemolysis, renal insufficiency, cholestatic hepatic dysfunction and iron overload [3].

Trans annular plane systolic excursion (TAPSE) has been noted as a validated marker for right ventricular systolic dysfunction and has been seen as an easily measurable parameter in evaluating right ventricular systolic function [4].

Elevated tricuspid regurgitation velocity (TRV), TAPSE and Tricuspid inflow (e/a) velocity (TV E/A) which are surrogate markers for pulmonary hypertension (PHT) are very relevant indices of right ventricular dysfunction which occurs in children with sickle cell anaemia and is associated with low hemoglobin and elevated reticulocyte count [5].

Accurate assessment of right ventricular function of children with sickle cell anaemia in steady state by echocardiography helps in early detection of cardiac diseases, enhances risk stratification, and allows timely initiation of appropriate therapy [6].

An assessment of right ventricular function among children with sickle cell anaemia is known to have some predictive values for deteriorating clinical outcome [7]. For instance, increases in right ventricular diastolic and systolic dysfunction have been reported to correlate with frequency of acute chest syndrome [7].

Assessment of ventricular function in children with sickle cell anaemia in steady state using echocardiography can assist in early detection of right ventricular dysfunction and pulmonary hypertension [7]. Treatments such as use of drugs that enhance right ventricular function could potentially reverse the disease process as well as prevent the increased morbidity and mortality associated with them [7].

Few studies on right ventricular function among children with sickle cell anaemia have been documented. For instance, Barakat [8] et al., in Nigeria, documented an increase in chamber dilatation and ejection fraction in subjects when compared to control. However, the study failed to use TAPSE and tricuspid inflow velocities to assess right ventricular dysfunction. These indices (TAPSE and tricuspid inflow velocities) are better harbinger for detecting right ventricular dysfunction than chamber sizes. Matins [9] et al. in his study, in Brazil, noted significant difference in ventricular function of children with sickle cell anaemia, when compared to normal. They noted that this difference could be due to eccentric hypertrophy of the left ventricle.

The use of TAPSE and Tricuspid inflow (e/a) velocity (TVE/A) in assessing right ventricular function is not commonly used in this setting, this also creates a yearning gap in determining the actual ventricular function in children with SCA as the traditional use of chamber size and dimension have lots of flaws and false positives.

Apart from assessing right ventricular function (Systolic and diastolic), this study went further to ascertain the prevalence of right ventricular dysfunction (Systolic and diastolic) which is not usually documented in many studies among children in Nigeria.

The use of TAPSE and tricuspid inflow (e/a) velocity (TVE/A) have better predicting effects on right ventricular systolic and diastolic dysfunction respectively when compared with other methods such as M mode and myocardial contractility [8]. Published works on cardiac changes in SCA are also limited in Africa [9]. This work could also form a template for future studies.

Detecting those with right ventricular dysfunction and pulmonary hypertension would aid in early intervention. This intervention includes starting on drugs that modulate and enhance right ventricular systolic and diastolic function and as such, improve quality of life of these children.

The study is therefore aimed at determining the mean Trans annular plane systolic excursion (TAPSE) in millimeter, mean right ventricular systolic pressure (RVP) in mmHg and mean tricuspid inflow velocity in (TV E/A) in children with sickle cell anemia in steady state compared with age and sex matched controls with haemoglobin AA genotype, it also seeks to ascertain the prevalence of right ventricular systolic dysfunction using TAPSE and the correlation between TAPSE, TVE/A and tricuspid regurgitant velocity (TRV) in subjects and controls. Moreover, the study is aimed at determining the prevalence of pulmonary hypertension using mean tricuspid regurgitant velocity (TRV) in m/s and the prevalence of right ventricular diastolic dysfunction using tricuspid inflow velocity (TV E/A) in children with sickle cell anemia compared with that obtained in age and sex matched controls with haemoglobin AA genotype, the work is also aimed at determining the relationship between age, gender and mean Trans annular plane systolic excursion and mean Tricuspid inflow (e/a) velocity among children with sickle cell anaemia compared to control.

## Methods

### Study design

This was a descriptive, cross-sectional, comparative study that assessed the right ventricular function among children with sickle cell anaemia in Enugu. Children with sickle cell anaemia who attended the sickle cell clinic and fulfilled the inclusion criteria were consecutively recruited into the study. The controls were apparently healthy HbAA children as determined by Hb electrophoresis, matched for age and sex who were attending follow up at the consultants’ clinic or children out-patient clinic.

Anthropometric measurements such as weight, height, body surface area and body mass index were taken in both subjects and controls, with the above measurements, Z scores of all indices of RV function were calculated.

The questionnaire consists of demographic variables, echocardiographic measurements of cardiac structures and functions especially right ventricular function.

Normal value of TAPSE was taken as 0.9-30 mm, normal tricuspid regurgitant velocity in m/s, was taken as < 2.5 m/s and normal right ventricular systolic pressure was taken as < 25 mmHg while normal right ventricular diastolic dysfunction (TV E/A) was taken as 0.8 to less than 2.1.

Pulmonary hypertension (mmHg) was calculated by adding the value of tricuspid regugitant velocity in m/s to right atrial pressure or central venous pressure (CVP) which is 10 mmHg [10].

Right ventricular diastolic function was ascertained using Tricuspid inflow velocity (TVE/A). The cursor was placed at the lateral aspect of the tricuspid valve annulus and the inflow velocity was then measured with a pulse wave. The early, rapid filling phase of diastole is represented by the E-wave. The E-wave deceleration time reflects right ventricular relaxation. Atrial contraction occurs in late diastole, and is represented by the A-wave [11].

TAPSE was obtained by placing the M-mode cursor through the lateral portion of the tricuspid valve annulus in the apical four-chamber view. The excursion of the tricuspid valve from the base of the heart towards the apex was measured as the distance from the annulus to the apex at end diastole minus that distance at end systole [11].

The prevalence of pulmonary hypertension was calculated as number of subjects or control with pulmonary hypertension divided by total number of subjects and control. In addition, the prevalence of right ventricular diastolic dysfunction was calculated using the same method. The prevalence of right ventricular diastolic dysfunction was calculated by the number of those with abnormal tricuspid inflow velocities divided by the total population of subjects and controls.

### Settings

This study was carried out in two tertiary hospitals, the University of Nigeria Teaching Hospital (UNTH), Ituku-Ozalla, Enugu, Nigeria and Enugu State University Teaching Hospital (ESUTH), Enugu. The University of Nigeria Teaching Hospital has a total bed space of 480 and provides specialized services in management of children with sickle cell anaemia and the hospital also serves as a referral centre for children with cardiac diseases. ESUTH is also a referral centre for children with sickle cell anaemia, their paediatric cardiac centre is in a budding phase.

University of Nigeria Teaching Hospital (UNTH) Ituku-Ozalla has the state of the art facility for management of all cases of cardiac diseases and performs open heart surgery in children. Enugu is in the South East geographic zone of Nigeria.

### Participants

These were children aged 3 years to 17 years, 11 months who attended the sickle cell clinics of the study hospitals, who were in steady state (Children in steady state are those with haemoglobin SS who are clinically stable for a minimum of 4 weeks and have not had blood transfusion for 3 months before recruitment [10]). The control population were children who were apparently healthy with haemoglobin genotype (HbAA) (matched for age and gender) and who came for follow up for common illnesses like malaria either in consultant clinic or children outpatient clinic.

The lower age limit of 3 years was chosen because the onset of right ventricular dysfunction in children with sickle cell anaemia is noticeable from the age of 3 years [3].

### Consent

A written consent was obtained from each parent/ caregiver of the subjects and controls after explaining to them, in detail, the objectives of the study as well as the echo procedure.

### Child assent

Assent was obtained from children older than seven years.

### Inclusion criteria for subjects

1. Children with sickle cell anaemia in steady state aged 3 years to 17 years, 11 months

### Exclusion criteria for subjects

1. Children with sickle cell anaemia who have congenital or acquired cardiac anomalies
2. Subjects who currently have sickle cell crises.
3. Subjects whose parents refused to give consent or children who refused assent to participating in the study
4. Subjects with previously corrected congenital heart diseases.

### Inclusion criteria for controls

Controls aged 3 years to 17 years, 11 months with Genotype AA who came for follow up at the children out patient or consultant clinic matched for age and sex.

### Exclusion criteria for controls

1. Children with congenital or acquired cardiac anomalies.
2. Controls whose parents refused to give consent or children who refused assent to participating in the study.
3. Children with previously corrected congenital heart diseases

Study Duration: The study was done over a four-month period (April 2019 to July 2019).

#### Study tool

The examinations were performed using the Hewlett-Packard (HP) model SONO 2000 Ultrasound Imaging System. The machine has a transducer with multi-frequency in the range 5.5-12 MHz for children, and this was used for the study. For each examination, the child was laid supine or on the left lateral decubitus position. For each patient, intra-cardiac anatomy was studied using the standard 2D echocardiographic views, right ventricular function was studied using TAPSE and tricuspid in flow velocities. All values obtained were converted to Z scores.

The study had a quality control where another cardiologist got his findings at certain intervals so as to reduce bias.

### Sample size estimation

The minimum sample size used in this study will be calculated using the formular [12].

$$ \mathrm{n}=\frac{\ {\left({\mathrm{Z}}_{\upalpha}+{\mathrm{Z}}_{1\hbox{-} \upbeta}\right)}^2\left({\mathrm{p}}_1\left(1\hbox{-} {\mathrm{p}}_1\right)+{\mathrm{p}}_2\left(1\hbox{-} {\mathrm{p}}_2\right)\right)}{{\left({\mathrm{p}}_1\hbox{-} {\mathrm{p}}_2\right)}^2} $$

Minimum sample size = 37.

Where n = minimum sample size in each group.

Z_α_ = standard normal deviate corresponding to 5% level of significance = 1.96.

Z_1-β_ = standard normal deviate corresponding to a power of 80% = 0.84.

P_1_ = proportion of right ventricular dysfunction in children with SCA based on the previous study, p_1_ = 0.20.

p2 = proportion of right ventricular function among normal children population; based on previous study, p_2_ = 0.45.

p1 – p2 = the smallest difference between the two groups of scientific or clinical n = (1.96 + 0.84)^2^ x {0.2(1–0.2) + 0.45(1–0.45)}/(0.45–0.20)^2^ = 37.

To obtain an appropriate sample size for 250 children on regular follow-up at UNTH Paediatric sickle cell clinic, the formula for determining sample size in finite population will be used.

$$ \frac{\mathrm{n}={\mathrm{n}}_0/1+\left({\mathrm{n}}_0-1\right)}{\mathrm{N}} $$

Where;

n = adjusted sample size.

n_0_ = calculated sample size.

N = population size

- $$ \mathrm{n}=\frac{\ 38}{1+\frac{\left(38\hbox{-} 1\right)}{250}} $$
  *n* = 35. 20% attrition rate will be used; this brings the final sample to 36 but rounded off to 50.

### Data analysis

Trans annular plane systolic excursion (TAPSE) and Tricuspid inflow (e/a) velocity was analyzed using Mean (SD). Mean TAPSE was compared using Student T test. Mean Trans Tricuspid inflow (e/a) velocity was compared using Student T test.

The relationship between age and mean Trans annular plane systolic excursion and mean Tricuspid inflow (e/a) velocity was ascertained using Pearson correlation variable. Proportion of children with abnormal right ventricular function was compared using Chi-square test and the proportion of subjects and controls who presented with pulmonary hypertension was ascertained using prevalence rate.

Level of significance was taken as *p* < 0.05.

## Results

Demographic characteristics of subjects and controls.

Table 1 shows the sex and age distribution of the participants.

**Table 1:** Demographic characteristics of subjects and controls Table I: Sex and age distribution of subjects and controls

		Sex				
		n %		χ 2	P	
Genotype						
SS	Male	28	54.9			
	Female	23	45.1	0.86	0.8	
	Total	51	100.0			
AA	Male	26	52.0			
	Female	24	48.0	0.85	0.7	
	Total	50	100.0			
**Age**		Mean	n	%	t	p
SS	Preschool	4.11 ± 0.78	9	17.6		
	School age	8.46 ± 1.28	24	47.1		
	Adolescence	14.22 ± 2.56	18	35.3		
	Total	9.73 ± 4.09	51	100.0	1.05	0.3
AA	Preschool	4.21 ± 0.89	14	28.0		
	School age	8.05 ± 1.43	19	38.0		
	Adolescence	13.65 ± 1.45	17	34.0		
	Total	8.88 ± 4.00	50	100.0		

χ 2 = Chi-square, t = Students t test, p = probability, preschool (3–5 years), school age (6–10 years), adolescence (≥11 years)

The mean age of subjects was 9.73 ± 4.09 while that of the controls, was 8.88 ± 4.00. There was no significant difference between the mean age of subjects (9.73 ± 4.09) and controls (8.88 ± 4.00) {t = 1.05, *p* = 0.3). The mean age of both male and female subjects (9.75 ± 3.98 years and 9.83 ± 4.60 years respectively) were similar {t = − 0.62, *p* = 0.95} and this was similar to the findings among the control group where there was no difference between the mean age of males (8.81 ± 3.91) and females (8.96 ± 4.18) {(t = − 0.13, *p* = 0.89} The Male: Female ratio was 1:1.

### Mean TAPSE, right ventricular pressure and tricuspid in flow velocities of subjects and controls

Both mean TAPSE and mean right ventricular pressure (RVP) were significantly higher in subjects than controls (28.24 ± 5.23 (Zscore: 0.258 ± 1.10)), (21.61 ± 9.49 (Zscore: 0.49 ± 1.2)) vs (25.82 ± 3.59 (Zscore: - 0.263 ± 0.80)), (13.89 ± 2.10 (Zscore: − 0.49 ± 0.3)) {t = 2.703, *p* = 0.008, t = 5.613, < 0.001}. The adolescent age group had higher values of TAPSE than the school aged and the preschool aged children in both subjects and controls (30.88 ± 4.71, 27.73 ± 4.68, 24.29 ± 5.18 respectively) vs (28.44 ± 2.30, 25.26 ± 3.00, 23.40 ± 3.65 respectively). There was moderate positive correlation between age and TAPSE in both subjects and controls (Pearson correlation coefficient = 0.5, *p* < 0.001 and 0.6, *p* < 0.001 respectively). In contrast to TAPSE, RVP was higher in the preschool than in school aged children and the adolescents in both subjects and controls (22.54 ± 10.73, 22.38 ± 8.26, 20.14 ± 10.65 respectively) vs (15.01 ± 2.55, 13.69 ± 0.75, 13.19 ± 0.99 respectively). There was a negative correlation between age and RVP in both subjects and controls, although the correlation was significant only in controls (coefficient = − 0.1 vs − 0.36, *p* = 0.5 vs 0.01).

As depicted in Table 2, the mean tricuspid regurgitant velocities in m/s of subjects, 1.88 ± 1.43 (Zscore: 1.87 ± 1.4) was higher than that in control, 1.70 ± 0.21 (1.69 ± 0.2) but the difference in mean was not found to be statistically significant, (Student t = 0.904, *p* = 0.34), Among the different age groups, the mean tricuspid regurgitant velocities in subjects were 1.79 ± 0.39, 1.66 ± 0.19, 2.22 ± 2.43 for preschool, school aged and adolescents respectively while for controls, it was 1.65 ± 0.15, 1.73 ± 0.19, 1.68 ± 0.27 respectively.

**Table 2:** Mean TAPSE, right ventricular pressure, and tricuspid in flow velocities in subjects and controls

Variable	Subjects	Controls	t	p
TAPSE	28.24 ± 5.23	25.82 ± 3.59	2.703	0.008
TRV	1.88 ± 1.43	1.70 ± 0.21	0.90	0.37
TVE/A	21.61 ± 9.49	13.89 ± 2.10	5.61	< 0.001

*TRV* (Tricuspid regurgitant velocity), *TAPSE* (tricuspid annular plane systolic excursion), *TVE/A* (Tricuspid inflow velocity)

### Prevalence of right ventricular systolic dysfunction using TAPSE

Table 3 shows proportion of subjects and controls with right ventricular dysfunction. Greater proportion of subjects had abnormal TAPSE (TAPSE above or below 2-SD from the mean of standard population, based on age of participant), 4 (8.0%) when compared with the control, 1 (2.0%).

**Table 3:** Prevalence of right ventricular dysfunction using TAPSEss

Genotype SS		Genotype AA		
TAPSE	N	%	N	%
+2SD	^a^3	6	0	0
+1SD	7	14	3	6
>− 1 to < +1SD	25	50	39	78
-1SD	14	28	7	14
-2SD	^a^1	2	1	2
Total	50	100	50	100

Abnormal TAPSE is value ±2SD from the mean of standard population, calculated with Echo z-score calculator, ^a^abnormal TAPSE

### Prevalence of pulmonary hypertension

A significantly higher proportion of subjects (25.5%) had Pulmonary hypertension (RVP in mmHg) when compared with control (2.0%) and the difference in proportions was found to be statistically significant, (χ^2^ = 11.668, *p* = 0.001). The prevalence of right ventricular diastolic dysfunction (Abnormal TV E/A)) in subjects was 9.8% while control was 0%.

### Indices of RV function in subjects based on gender

With respect to gender, 14.3% of male subjects had pulmonary hypertension (RVP), compared to 4.3% females (χ^2^ = 1.410, *p* = 0.235).

For subjects, the mean TAPSE in millimetre for males was 28.12 ± 5.84 (Zscore: TAPSE; 0.23 ± 1.26) and this was lower than that for females, 28.37 ± 4.51 (Zscore: 0.29 ± 0.97) but the difference in mean was not found to be statistically significant, (t = − 0.170, *p* = 0.866). The mean TVR for males, 17.87 ± 8.32 (Zscore: − 0.21 ± 0.92) was higher than that for females, 16.79 ± 2.98 (Zscore: − 0.27 ± 0.74) but the difference in mean was found not to be statistically significant, (t = 0.27, *p* = 0.79). The mean TVE/A for males, 2.06 ± 1.93 (Zscore: 0.26 ± 1.89) was higher than that for females, 1.67 ± 0.22 (Zscore: − 0.11 ± 0.22) but the difference in mean was found not to be statistically significant, (t = 0.94, *p* = 0.35)-Table 4**.**

**Table 4:** Indices of RV function in Subjects based on gender

Variable	Male (*n* = 27)	Female (*n* = 23)	Student t	*p* value
TAPSE				
Mean (±SD)	28.12 ± 5.84	28.37 ± 4.51	0.170	0.90
Zscore: TAPSE	0.11 ± 1.10	−0.11 ± 0.90	0.13	0.90
TRV + 10				
Mean (±SD)	17.87 ± 8.32	16.79 ± 2.98	0.578	0.57
TVE/A				
Mean (±SD)	2.06 ± 1.93	1.67 ± 0.22	0.942	0.35
Zscore: TVE/A	0.07 ± 1.40	−0.08 ± 0.2	0.8	0.40

SD (standard deviation) TRV + 10 (Tricuspid regurgitant velocity); TAPSE (tricuspid annular plane systolic excursion); TVE/A (Tricuspid inflow velocity)

### Indices of RV function in controls based on gender

For Control, the mean TAPSE in millimetre for males, 25.99 ± 3.71 (Zscore: − 0.23 ± 0.80) was higher than that of females, 25.64 ± 3.52 (Zscore: − 0.30 ± 0.76) but the difference in mean was not found to be statistically significant, (t = 0.34, *p* = 0.74). The mean TVR for males, 14.54 ± 1.83 (Zscore: 0.29 ± 1.11) was comparable to that of females, 13.77 ± 1.25 (Zscore: 0.18 ± 1.10), (t = 0.38, *p* = 0.71). The mean TVE/A for males, 1.66 ± 0.25 (Zscore: − 0.12 ± 0.24) was lower than that of females, 1.73 ± 0.16 (− 0.05 ± 0.16) but the difference in mean was not found to be statistically significant, (t = − 1.11, *p* = 0.26)-Table 5**.**

**Table 5:** Indices of RV function in controls based on gender

Variable	Male (*n* = 27)	Female (*n* = 23)	Student t	*P* value
TAPSE				
Mean (±SD)	25.99 ± 3.71	25.64 ± 3.52	0.34	0.74
Zscore	− 0.23 ± 0.80	− 0.30 ± 0.76	0.34	0.74
TRV				
Mean (±SD)	14.54 ± 1.83	13.77 ± 1.25	1.73	0.09
Zscore	0.29 ± 1.11	0.18 ± 1.10	0.38	0.71
TVE/A				
Mean (±SD)	1.66 ± 0.25	1.73 ± 0.16	1.11	0.27
Zscore	−0.12 ± 0.24	−0.05 ± 0.16	−1.11	0.26

*TRV* (Tricuspid regurgitant velocity), *TAPSE* (Tricuspid annular plane systolic excursion), *TVE/A* (Tricuspid inflow velocity)

### Correlation of age with indices of RV function of TAPSE and TVE/a in subjects and controls

There was a strong positive correlation between age in years and TAPSE in millimetre in both subjects and controls, increases in age were correlated with increases in TAPSE in millimetre and this was found to be moderately statistically significant (*n* = 50, *r* = 0.52, *p* < 0.001) and (*n* = 50, *r* = 0.62, *p* < 0.001) respectively. There was no correlation between age and TVE/A in subjects and controls (*n* = 50, *r* = 0.05, *p* = 0.75) and (*n* = 50, *r* = 0.06, *p* = 0.66) respectively.

## Discussion

We noted a significant difference in right ventricular systolic function among children with sickle cell anaemia when compared with control, although all values remain within normal reference range. On further analysis, 8% of subjects have right ventricular systolic dysfunction (Using TAPSE) compared to 2% noted in control. These results are similar to other reports which also demonstrated right ventricular systolic dysfunction in spite of cardiac dilatation among children with Haemoglobin SS [4–9]. The prevalence of right ventricular systolic dysfunction seen in this study was lower than that obtained by Simbo et al. who had a prevalence of 39% [13]. The reason for the lower prevalence was that the authors used a very large sample size; again their study was a retrospective review of previous echocardiogram done among children with sickle cell anaemia.

When we stratified right ventricular function by means of TAPSE by age, we found that TAPSE was higher among adolescents than school and preschool groups. This can be explained by increase in stoke volumes and decease in heart rate seen in older children [14, 15]. The reverse is the case for tricuspid velocity. Tricuspid velocity is higher in preschool than school and adolescent children. This is due to the fact that increase peripheral vascular resistance which causes increases in tricuspid velocity is commoner in younger ages [15, 16].

It is important to note from this study, that though the tricuspid regurgitation gradient which is a surrogate of RV systolic function and pulmonary hypertension were higher in children with sickle cell anaemia compared to those with haemoglobin AA genotype, yet all values fell within normal reference range. These findings may show that children with sickle cell anaemia have a greater tendency of having pulmonary hypertension. We noted that the prevalence of pulmonary hypertension (PHT) using tricuspid regurgitation (TR) velocity gradient of more than 2.5 m/s and right ventricular pressure (RVP) of more than 25 mmHg, among subjects and controls was 25.5 and 2% respectively. The 25.5% prevalence obtained in our study is similar to 22.3% obtained by Sokumbi et al. [17]. Our findings however are similar to that obtained in a study in Northern Nigeria, where a prevalence of 25% was obtained [18].

There is paucity of studies on the prevalence of PHT among children with sickle cell anaemia especially in developing countries and thus its significance in this age group is not well established [9]. Whereas there are studies on children from western countries which revealed prevalence ranging from 20 to 33% [16, 17] extrapolating these findings to the paediatric population in African countries may be misleading.

In children with sickle cell anaemia, estimated pulmonary systolic pressure correlates well with measurements obtained by cardiac catheterization [9]. A value of 2.5 m /s or more corresponds to an estimated pulmonary artery systolic pressure of 35 mmHg [9]. While some authors have defined PHT as TRV of 3.0 m/s or more, values of at least 2.5 m /s have been associated with an increased risk of death among children with sickle cell anaemia [9]. The prevalence of pulmonary hypertension seen in this study is higher than that obtained by Adedoyin et al. [9] who obtained a prevalence rate of 3.6% among children with sickle cell anaemia compared to none in controls. The lower prevalence obtained by Adedoyin could be because the sample frame was from younger ages and the cases were a selection of patients who were assumed to be better motivated for regular treatment in a tertiary hospital and in a commercial centre of the country.

Qurechi et al. [3], in his study among children, using TR gradients > 2.5 m/sec, consistent with pulmonary hypertension, noted a prevalence rate of 16% among children with sickle cell anaemia which is lower than 25.5% obtained in our study. Qureshi’s finding is different from ours in that they studied children older than 9 years whereas our study involved children in the age bracket 3–17 years [3]. However age alone may not necessarily explain this difference.

It is important to note that this increase of Tricuspid velocity (a surrogate of pulmonary hypertension) and TAPSE could be caused by increased TRV with severe haemolysis, elevated right ventricular filling pressure, renal dysfunction, and high circulating erythropoietin concentrations especially among children with sickle cell anaemia [19]. It is also suggested that children with sickle cell anaemia have greater tendency of having RV dysfunction because they have higher circulating erythropoietin concentrations [20]. .This could also be a marker of the potential contributions of increased erythropoiesis to increased pulmonary artery pressure [21].

In summary, the prevalence of pulmonary hypertension varies between studies and the frequency rises with age; however, the peak age of occurrence of PHT varies from one region to another. The variation in prevalence could also be a reflection of the different phenotypic expression of haemoglobin SS which presents with various degrees of organ damage and survival.

Pulmonary hypertension is a life-threatening complication among children with sickle cell anaemia and may be clinically silent until late in the course of the disease [22]. It has been linked to accelerated mortality as mortality may be as high as 40% [23] or 10-fold higher compared with those with normal TRV (Surrogate of pulmonary hypertension) [23]. This study showed that TAPSE is a better predictor of right ventricular systolic dysfunction when compared with TRV where results showed proportion of right ventricular dysfunction as 50 and 25.5% respectively [23].

Right ventricular diastolic dysfunction is a mechanical abnormality that is caused by breakdown in the passive compliance and active myocardial relaxation; an intrinsic property of the ventricle during diastole [24]. Diastolic dysfunction in children with sickle cell anaemia could be due to a pathological state that adversely affect the passive compliance during diastole, such as increases in myocardial wall thickness observed in concentric hypertrophy cardiomyopathy as a result of longstanding ischemia or hypoxaemia [25–28].

When we looked at the overall prevalence rate of right diastolic dysfunction for both subjects and controls, we noted that children with sickle cell anaemia had a prevalence rate of 9.8% compared with their normal counterparts who had 0% prevalence. One study has also implicated a high prevalence of diastolic dysfunction of 45% among subjects with SCA [22].

Regrettably, the author used Pulse wave diameter (PWD) and tissue Doppler index (TDI) to assess RV dysfunction. This indeed explains the differences between outcome of our study and theirs. TVE/A is superior to TDI indices because it is reproducible, easy to read and not prone to bias.

It is noteworthy to point out that diastolic dysfunction and pulmonary hypertension contribute independently to mortality in children with sickle cell anaemia. Children with both risk factors have extremely poor prognosis. These data support the implementation of echocardiographic screening of children with sickle cell anaemia in the attempts to identify high-risk individuals for further evaluation [4–15].

We noted significant increases in TAPSE and TVE/A with increasing age in both subjects and controls showing that both functions worsens as age progresses. It is important to note that in children with sickle cell anaemia, aging is associated with increased levels of haemolysis, increased intimal proliferation, narrowing of pulmonary vessels and eventual increase of wedge pressure with attendant increase in pulmonary vascular resistance. These could then lead to alterations in right ventricular pressure.

However, we noted no significant association between age and pulmonary hypertension. Shokubi et al. [17] also noted no association of age/gender and indices of right ventricular dysfunction in their study. These findings were also in keeping with outcome of other studies conducted in the United States of America [18].

## Conclusion

The mean TAPSE of children with sickle cell anaemia was significantly higher than that obtained in children with haemoglobin AA genotype. The mean tricuspid inflow velocities (TVE/A) of children with sickle cell anaemia was higher than that gotten in children with controls. The prevalence of right ventricular dysfunction among children with sickle cell anaemia is 50% compared with 22% obtained in control. These increase in right ventricular systolic and diastolic functions in subjects showed that these children have more tendency of right ventricular systolic and diastolic dysfunction. The prevalence of pulmonary hypertension is higher in subjects compared to controls. Increase in indices of right ventricular function was influenced by age but independent of gender.

### Recommendations

Children with sickle cell anemia in steady state have high tendency of developing right ventricular dysfunction when compared with their counterparts with hemoglobin AA genotype. It is therefore recommended that: Indices of right ventricular function should be assessed routinely among children with sickle cell anaemia. Indices of right ventricular function among children with normal haemoglobin genotype may be used as normative values especially in this locale.

### Strength of the study

The study is a cross sectional comparative study and prospective in nature. Again this is the first time this type of work is done in Enugu. It may therefore provide baseline values that could be useful in subsequent studies. The instrument (echocardiography) used was validated.

### Limitations

This is hospital based study and thus generalization to the community may be difficult.

## Data Availability

The data will not be shared in order to protect the participants’ anonymity*.*

## Abbreviations

TAPSE: Trans annular plane systolic excursion
RVP: Right Ventricular Pressure
TVE/A: Tricuspid valve inflow velocities
ACS: Acute chest syndrome
TRV: Tricuspid regurgitant Velocity
RV: Right Ventricle
TDE: Tissue Doppler echocardiography
HBAA: Haemoglobin AA genotype
ESUTH: Enugu State University Teaching Hospital
MHZ: Megahertz
PHT: Pulmonary Hypertension
PWD: Pulse Wave diameter
TDI: Tissue Doppler Imaging
